# Genotype diversity in the honey bee parasite *Nosema ceranae*: multi-strain isolates, cryptic sex or both?

**DOI:** 10.1186/s12862-016-0797-7

**Published:** 2016-10-18

**Authors:** Soledad Sagastume, Raquel Martín-Hernández, Mariano Higes, Nuno Henriques-Gil

**Affiliations:** 1 Centro Apícola Regional, Bee Pathology Laboratory, 19180 Marchamalo, Guadalajara, Spain; 2 Instituto de Recursos Humanos para la Ciencia y la Tecnología (INCRECYT), Parque Científico de Albacete, Spain; 3 Departamento de Ciencias Médicas Básicas, Facultad de Medicina, Universidad CEU San Pablo, Campus de Montepríncipe, 28668 Madrid, Spain

**Keywords:** Heterozygosis, Honeybee parasite, Microsporidia, Populations, Recombination, Sexual reproduction, Single nucleotide polymorphism

## Abstract

**Background:**

There is great controversy as to whether Microsporidia undergo a sexual cycle. In the paradigmatic case of *Nosema ceranae*, although there is no morphological evidence of sex, some meiosis-specific genes are present in its reduced genome and there is also high intraspecific variability, with incongruent phylogenies having been systematically obtained. The possibility of sexual recombination is important from an epidemiological standpoint, particularly as *N. ceranae* is considered to be a major factor in the current disquieting epidemic of widespread bee colony losses. This parasite apparently originated in oriental honey bees, spreading out of Asia and Australia to infect honey bees worldwide. This study had three main objectives: i) to obtain genetic markers that are not part of known multi-copy arrays for strain determination; ii) to shed light on the intraspecific variability and recombination of *N. ceranae*; and iii) to assess the variability in *N. ceranae* populations. The answers to these questions are critical to understand the capacity of adaptation of microsporidia.

**Results:**

Biallelic polymorphisms were detected at a number of specific points in the five coding loci analyzed from European and Australian isolates of *N. ceranae*. Heterozygous genotypes were abundant and cloning experiments demonstrate that they reflect the existence of multiple alternative sequences in each isolate. The comparisons of different clones and genotypes clearly indicate that new haplotypes are generated by homologous recombination.

**Conclusions:**

The *N. ceranae* isolates from honey bees correspond to genotypically distinct populations, revealing that individual honey bees may not be infected by a particular clone but rather, a pool of different strains. Homologous recombination implies the existence of a cryptic sex cycle yet to be described in *N. ceranae*. There are no diagnostic alleles associated with Australian or European origins, nor are there differences between the two hosts, *A. cerana* and *A. mellifera*, supporting the absence of biological barriers for *N. ceranae* transmission. Diversity is high among microsporidia of both these origins, and the maintenance of a high heterozygosis in the recently invaded European populations, could hypothetically underlie the stronger virulence of *N. ceranae* observed in *A. mellifera*.

## Background

Microsporidia are obligate intracellular parasites classified in the Kingdom Fungi [1, 2] and recently associated with Cryptomycota [3]. Unicellular and eukaryotic, these spore parasites lack some cellular organelles and they have a reduced genome [4]. More than half of the known Microsporidia genera possess a diplokaryotic nuclear apparatus, at least during some stages of their life cycle [5], and the two apposed nuclei are assumed to be identical and divide synchronously [6]. Mostly, microsporidia reproduce clonally, however, increasing evidence of occasional sex has important consequences from an epidemiological standpoint [5, 7–9].

*Nosema ceranae* was first described as a microsporidium parasite of the Eastern honey bee *Apis cerana* [10], yet in the last decade it has gained importance due to its worldwide presence in a new host, *Apis mellifera*, the European honey bee [11, 12]. Significantly, this host has suffered a dramatic phenomenon of colony collapse around the world. Within the individual host, the life cycle of *N. ceranae* is completed in only 3 days, by which time new mature infective spores are ready to be released [13]. A number of studies point out that the parasite comes originally from oriental honeybees and has recently been transmitted worldwide [14]. Hence, the genetic variability among *N. ceranae* populations could have aided its ongoing spread out of Asia and Australia, driving higher diversity in Eastern populations and local bottlenecks.

In recent years the search for genetic markers that are useful in phylogenetic studies and strain determination of this microsporidium has proved to be more difficult than initially thought. The first choice as genetic marker are usually the ribosomal genes and spacers (rDNA), yet these gene clusters are not only repeated but they are also variable [7, 15–17]. In *N. ceranae,* the ribosomal small subunit (SSU) and the intergenic spacer (IGS) display strong polymorphism, and recombination creates new variants, compromising genotyping and suggesting sexual reproduction [18]. Recently, some putatively single copy genes have proved to be polymorphic in this species [19–23], again suggesting recombination. Although recombination is not an absolute proof of sex, it was demonstrated that the detection of genetic diversity, recombination and horizontal gene transfer in Microsporidia strongly suggest an extant sexual cycle during the infection of these organisms [9].

In fungi, the wide array of reproductive strategies in natural populations speeds up adaptation and evolution [24]. Nevertheless, the hypothesis of sexual reproduction of *N. ceranae* remains somewhat controversial. No mono-nucleated forms have been described in its life cycle providing evidence of meiosis and the absence of the *Spo11* gene could suggest asexuality, although a number of meiosis-specific genes have been described in this microsporidium, such as *DMC1* [25]. Conversely, there is no data available regarding their karyotype and the ploidy of both nuclei has been recently questioned, leading to the hypothesis of high intragenomic variability in an asexual *N. ceranae* [26]. However, the maintenance of meiotic machinery in a reduced genome, the unclear phylogenies and the high intraspecific variability with no signs of concerted evolution, together with evidence of recombination, inevitably suggest this parasite undergoes sexual reproduction.

In the light of these facts, all of them suggesting the existence of cryptic sex, it becomes interesting to determine whether the two nuclei in *N. ceranae* are identical and whether all the cells in each isolate are genetically equivalent. Indeed, it is suspected that different strains exist in each *N. ceranae* isolate, reflecting genetically diverse populations [27]. Moreover, how and why there is such high variability in *N. ceranae* has also to be explained. Accordingly, our first goal was to obtain markers that are not part of known multicopy arrays and that are therefore useful for strain determination. These markers should help shed light on the intraspecific variability and recombination in *N. ceranae* (multicopy sequences may create new alleles after unequal sister chromatid exchanges) [18]. In addition, we assessed the variability in *N. ceranae* populations and localities, which is essential to understand the adaptability of this microsporidium.

To address these objectives, we analyzed five long randomly selected, purportedly unique fragments of coding DNA, not tandemly repeated genes (unlike ribosomal DNA), made available through the *N. ceranae* genome project [28]. We find that the use of long DNA fragments (e.g., approx. 1000 bp) provides more accurate information about natural genetic diversity and recombination than studies based on small fragments; using a massive sequencing technique, the capacity to detect LD is reduced to fragments down 200 bp, as the probability of recombination between two points increases with the distance. These DNA fragments were studied in different isolates from Europe, Asia and Australia. Given the Eastern origin of *N. ceranae*, and that *A. mellifera* and *A. cerana* co-exist in Australia, the information from Australian samples should be closer to the primary sources of *N. ceranae* and thus, can be compared with the data from the European samples.

## Methods

The microsporidium *Nosema ceranae* isolated from 30 biological samples was analyzed, including: the Western honey bee, *Apis mellifera* (*n* = 24); the Eastern honey bee, *A. cerana* (*n* = 5); and one regurgitated pellet with *N. ceranae* spores from the bee-eater, *Merops apiaster* (this latter sample was interesting because it was expected to contain the remains of many different bees captured by the bird). The samples and their locations are indicated in Table 1. Total DNA was extracted from the samples following the method described by Martín-Hernández et al. [29] and the total DNA from each of the 30 samples was considered an “isolate”. Polymerase Chain Reactions (PCRs) were performed to amplify 5 different fragments from randomly selected coding regions of *N. ceranae* with the following criteria: each was supposed to be single-copy loci, of at least 990 bp in order to obtain long amplicons ranging from 873 to 1280 bp, and should not include microsatellites (the GenBank references and the corresponding names of the loci are shown in Table 2). No significant matches with other organisms were obtained for any of these genes using the BLAST tool (http://blast.ncbi.nlm.nih.gov/Blast.cgi). The PCR mixtures included: 0.5 μL of the template DNA solution, 0.4 μM of each nucleotide, 10 pmol of each primer, 1.5 U of Platinum *Taq* DNA Polymerase (Invitrogen, cat. no.11509-015), its 10x buffer, 25 nmol MgCl_2_, 12 μg of BSA (Roche Diagnostic, cat. no. 10711454001) and sterilized distilled water to a final volume of 25 μL. PCRs were performed on an Eppendorf Mastercycler EpGradient S and a Pro S thermocycler following the program: 94 °C for 2 min; 40 cycles of 94 °C for 30 s, the primer-specific annealing temperature for 30 s, 68 °C for 60 s (for products under 1000 bp) or for 90 s (for products over 1000 bp); and a final elongation step at 68 °C for 7 min. The five different primer pairs, their sequences, the PCR product sizes and the correspondent annealing temperatures are also shown in Table 2. The PCR products were kept at 4 °C and 5 μL of each were ran in standard 2 % Agarose gel (Invitrogen E-GEL 2 % Agarose GP, cat. no. G8008-02) and visualized by Ethidium Bromide staining. PCR products were purified using QIAquick PCR Purification Kit (QIAGEN, cat. no. 28104) before sequencing in both directions at the Unidad de Biología Molecular (Universidad de Alcalá) using the same PCR primers (Table 2).

**Table 1:** Isolates of *Nosema ceranae* with their correspondent geographical origin, year of sampling and biological source

Isolate	Location	Year	Biological Source
Sp801	Ciudad Real, Spain	2005	*Apis mellifera*
Sp816	Cáceres, Spain	2005	*Apis mellifera*
Sp889	Cuenca, Spain	2005	*Apis mellifera*
Sp1103	Castellón, Spain	2005	*Apis mellifera*
Sp906	Guadalajara, Spain	2006	*Apis mellifera*
Sp2106	Alicante, Spain	2006	*Apis mellifera*
Sp402	Badajoz, Spain	2006	*Apis mellifera*
Sp610	Valencia, Spain	2006	*Apis mellifera*
SpN	Guadalajara, Spain	2006	*Apis mellifera*
SpTF	Guadalajara, Spain	2010	*Apis mellifera*
Sw479	Liebefeld, Switzerland	2006	*Apis mellifera*
G991	Freiburg, Germany	2006	*Apis mellifera*
F1109	Surgères, France	2006	*Apis mellifera*
SLO7	Ljubljana, Slovenia	2006	*Apis mellifera*
Sk807	Devínska Nová Ves, Slovakia	2008	*Merops apiaster*
A2	Queensland, Australia	2006	*Apis mellifera*
A3	Queensland, Australia	2006	*Apis mellifera*
A4	Queensland, Australia	2006	*Apis mellifera*
A5	Queensland, Australia	2008	*Apis mellifera*
A6	Queensland, Australia	2008	*Apis mellifera*
A10	Queensland, Australia	2010	*Apis mellifera*
A11	Queensland, Australia	2010	*Apis mellifera*
A13	Queensland, Australia	2004	*Apis mellifera*
A14	Queensland, Australia	2005	*Apis mellifera*
A22	Queensland, Australia	2010	*Apis mellifera*
A7	Queensland, Australia	2009	*Apis cerana*
A8	Queensland, Australia	2010	*Apis cerana*
A12	Queensland, Australia	2009	*Apis cerana*
A18	Queensland, Australia	2010	*Apis cerana*
A19	Queensland, Australia	2009	*Apis cerana*

**Table 2:** Primers and PCR data

Primer	Sequence (5’ – 3’)	Tm (°C)	Product size (bp)	GB reference/locus
X639_up	GTTAAAAACTGGGATATTCA	55.0	1280	XM_002996639 NCER_100183
X639_low	ACAAATCTATCTCTTATCCCT			
X580_up	AAGGGAAATATTAGGCAACTG	56.6	1138	XM_002996580 NCER_100253
X580_low	CGGGAAGTTCAATTACACTC			
X802_up	TGCCGAGTGATAAACTTACT	58.0	873	XM_002996802 NCER_100040
X802_low	AATCGATATATCTGCGTTCTT			
X754_up	ATGGCTTCTACAATTTACTTA	55.0	1123	XM_002996754 NCER_100059
X754_low	AAAACATATTCGTGCACTA			
X696_up	ATTTGAAATTGTCTCCCTATG	55.0	1217	XM_002996696 NCER_100117
X696_low	GGAAATTACTTCGTCAACCT			

Primers used in PCR and sequencing, PCR annealing temperature (°C), product DNA size (bp) and GenBank reference sequences, together with the correspondent locus name

In order to ensure that the variability found in the PCR products actually corresponds to distinct genomic DNA templates, the amplification products of 3 variable X639 fragments were selected (SpTF, Sp402 and Sp1103) and cloned into the *Escherichia coli* plasmid pCR2.1-TOPO® with the TOPO TA Cloning® Kit (Invitrogen, cat no. K4500-01). The plasmid DNA was extracted from randomly selected clones and purified using the QIAprep Spin Miniprep Kit (Qiagen, cat no. 27106), and it was digested (1 μg) with *Eco*RI (New England Biolabs, R0101S) and separated by 1 % agarose gel electrophoresis to check the correct size of the insert. A total of 38 clones were obtained (14, 6 and 18, respectively) and they were sequenced independently as described above. Additionally, the absence of recombination during the PCR amplification was tested as described elsewhere [30]. Briefly, two clones that differ by 6 bp were mixed, amplified with Platinum *Taq* polymerase, and the products were sequenced using M13 commercial primers. All the sequences obtained matched one of the two templates and no recombinants were detected.

All sequencing chromatograms were carefully revised visually, with the double-peaks noted as single nucleotide polymorphisms (SNPs) and identified with the code for the degenerate base positions (wobble positions IUB Code). The sequences obtained were aligned using the CLUSTAL W algorithm [31], and analyzed and translated into protein sequences with BIOEDIT 7.0.5.2 [32]. For a clearer study of the polymorphisms, common conserved areas were removed manually from the general alignments. The allele population frequencies p_i_ for a given variable site *i* was calculated assuming that double peak isolates are heterozygous and that those with a “clean” peak in the chromatogram are homozygous. The diversity for a site *i* with *j* alleles were estimated by D_i_ = 1– Σp_ij_^2^, and heterozygosis as the number of isolates with a double peak at the site *i* divided by the total number of isolates. Mean diversities were compared with standard t-student tests. The rates of synonymous (Ks) and non-synonymous substitutions (Ka) were calculated with the DNAsp 5.10 program [33], and sequence logo graphical representations were created with Weblogo 2.8.2 [34].

## Results

**Fig. 1:**
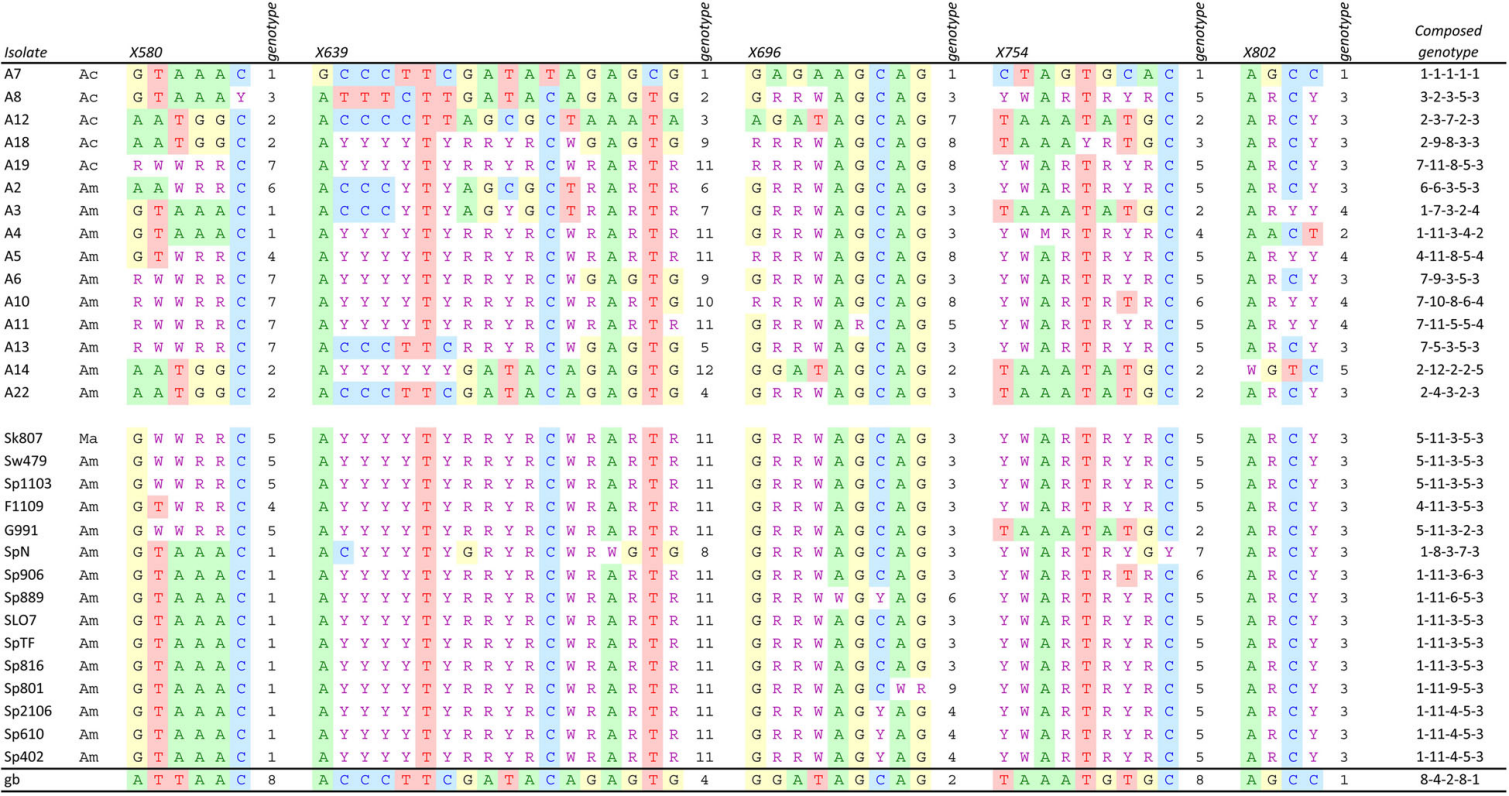
Genotypes obtained for the isolates of *N. ceranae* in the 5 markers analyzed. Only the variable sites are shown and the complete alignments are available in GenBank. The name of the isolate is followed by its correspondent origin: *Apis cerana* (Ac), *Apis mellifera* (Am) and *Merops apiaster* (Ma). Gb: Genbank reference sequences. The first 15 were Australian isolates and the last 15 European isolates. For each marker the sequence found in the isolate A7 was considered genotype 1, and the remaining were arbitrarily numbered. Nucleotides were named following the nomenclature of IUB code, so R = G or A, Y = C or T, W = A or T, and M = A or C

The five markers analyzed in the 30 *N. ceranae* isolates revealed sequences that were mostly identical to their corresponding GenBank references, although all of them contained some nucleotide variations. Figure 1 shows the polymorphic points which varied from 4 in X802 to 18 in X639. Compared to the reference sequence, the X580 marker showed 7 additional nucleotide differences, as well as a 3 bp indel; however, all of our sequences were identical so those differences were omitted from the analysis and they were not included in Fig. 1. The complete alignments can be seen at the GenBank references KJ544353-KJ544382, KJ544383-KJ544412, KJ544413-KJ544442, KJ544443-KJ544472 and KJ544473-KJ544502, for the X580, X639, X696, X754 and X802 sequences, respectively.

### Sequence polymorphisms

Only in one isolate (A7, obtained from *Apis cerana*, Australia) were all the sequences entirely free from double peaks (or indeterminate forms) and this was used for further comparisons. By contrast, unexpected nucleotide heterogeneity at certain points was found for all five markers, double peaks being evident on the direct sequencing chromatograms. The different isolates presented 2 to 31 indeterminate forms. The genotypes of the different isolates contained point substitutions, as well as the corresponding mixture or heterozygote. For instance, the variable sites in the case of X580 were GTAAAC in A7 and AATGGC in A12, while A19 was assigned RWWRRC as the sequencing systematically revealed clear double peaks at the first five sites.

**Fig. 2:**
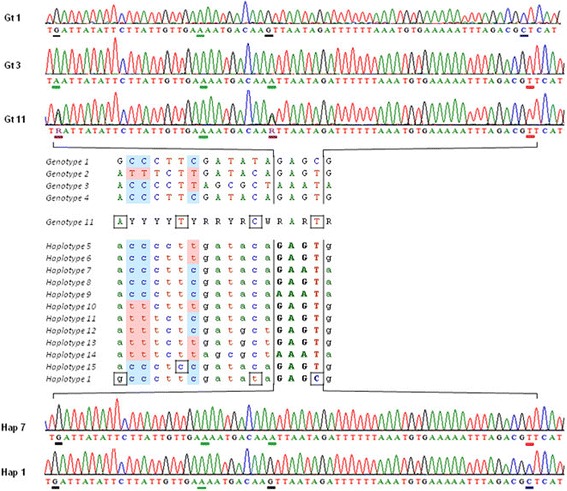
Partial comparison of direct sequencing results for X639 marker and cloned fragments. The chromatograms given above correspond to genotypes #1, #3 and #7, in the segment marked in the sequences of haplotypes (middle). Note that there are two double peaks (R) in Gt11 which are not ambiguous in Gt1 and Gt3 (G or A, but not both). Clones obtained from Gt11 had G or A in such points (below). Genotypes 1, 2, 3, and 4 were the only ones with no ambiguous points (see Fig. 1). Among the 38 clones analyzed, 15 haplotypes were obtained, including those identical to genotypes 1, 2, and 3, as well as several recombinant haplotypes and even two (#1 and #15) that were not expected from the sequence of genotype 11 and must represent minority sequences existing in the original isolate (see text). The nucleotides shadowed in the haplotypes correspond to the positions 159, 186 and 477 of the alignment

To ensure that the double peaks corresponded to different DNA templates in the *N. ceranae* isolates an additional experiment was performed. Thus, the original amplification products of three isolates (SpTF, Sp402 and Sp1103) were each cloned into *E. coli* plasmids and several clones were sequenced (see corresponding haplotypes at GenBank accessions KM042042 to KM042056). No double peaks appeared and indeed, the different clones included each of the variants that composed the genotype (for example, where a R existed in the original sequence, some clones had an A while others a G: Fig. 2). Thus, overlapping peaks are not artefacts but rather, they reflect the existence of more than one alternative sequence in each isolate. Likewise, in a second validation experiment performed as described previously [30], all the PCR products cloned fitted to one of the two haplotypes used as templates. Hence, in the conditions used for PCR amplification there was no in vitro recombination and thus, artefactual haplotypes were not generated.

However, 3 sequences obtained from the 38 clones did not match any of the possible variants predicted from the original sample. That is, at points where no indeterminate forms existed these clones had a different nucleotide (Fig. 2). The possibility of artefacts due to clone contamination was ruled out and although replication errors in the first PCR could be considered, it is important to note that those variants were not random but rather, they existed in separate samples. In fact, two of these clones exactly match a different genotype (#1). Therefore, in addition to the main types explained by a heterozygous *N. ceranae* strain, minority infection by genetically different strains may also occur. This also demonstrates that a given bee colony can be infected by more than one strain of *N. ceranae*.

All variations were biallelic, that is only two alternative nucleotides could be found for a given site plus the corresponding mixed or heterozygous genotype. As expected, the frequency of A/G transitions was similar to that of C/T (18 and 15 from a total of 46 SNPs), as a given transition in one DNA strand implies a complementary transition in the other. Yet surprisingly, no G/C (S) transversions were observed, in contrast to the 9 A/T (W) transversions at polymorphic sites. Thus, it seems that for some unknown reason *N. ceranae* is much more prone to weak transversions than to those at strong base pairs (Table 3 shows the number of variable sites and polymorphisms per site). All possible allelic variants were translated to the corresponding amino acid sequence using DNAsp software, obtaining the number of synonymous and non-synonymous changes, as well as the ratio between the polymorphisms at non-synonymous and synonymous sites (Table 4). Two loci (X580 and X802) only exhibited non-synonymous changes, in X639 there was an excess of synonymous substitutions, while the proportion Ka/Ks was compatible with a neutral model for X696 and X754.

**Table 3:** Polymorphism data for each marker

	Marker					
	X580	X639	X696	X754	X802	Mean
*N° of different genotypes*						
*Total (n = 30)*	7	12	8	7	5	7.6
*Europe*						
*Total (n = 15)*	3	2	3	4	1	2.8
*Australia*						
*Total (n = 15)*	6	11	6	6	5	6.6
*A. cerana (n = 5)*	4	5	4	4	2	3.8
*A. mellifera (n = 10)*	5	8	4	4	5	5.2
*Mean diversity*						
*Europe*						
*Total (n = 15)*	0.18	0.36	0.21	0.34	0.25	0.27
*Australia*						
*Total (n = 15)*	0.43	0.36	0.21	0.32	0.35	0.33
*A. cerana (n = 5)*	0.45	0.39	0.22	0.34	0.24	0.33
*A. mellifera (n = 10)*	0.41	0.34	0.21	0.35	0.33	0.33
*Mean heterozygosis*						
*Europe*						
*Total (n = 15)*	0.21	0.71	0.39	0.61	0.50	0.48
*Australia*						
*Total (n = 15)*	0.36	0.39	0.30	0.41	0.48	0.39
*A. cerana (n = 5)*	0.20	0.26	0.24	0.31	0.40	0.28
*A. mellifera (n = 10)*	0.61	0.56	0.38	0.67	0.54	0.55

**Table 4:** Polymorphism data per site

	*Marker*				
	*X580*	*X639*	*X696*	*X754*	*X802*
Total sites	1138	1280	1219	1123	875
Variable sites	6	18	9	9	4
Polymorphisms per site	0.0053	0.0141	0.0074	0.0080	0.0046
Synonymous	0	11	2	2	0
Non-synonymous	6	7	7	7	4
Ka/Ks	-	0.14	0.88	0.92	-

Variable sites for each marker of *N. ceranae*, polymorphisms per site, synonymous and non-synonymous changes, and the ratio between non-synonymous and synonymous rates (Ka/Ks)

### Geographical genotype distribution

Half of the *N. ceranae* isolates in this study were obtained from six different European countries and the other half were Australian isolates (Table 1). Thus, the number of different genotypes found for each sequence was evaluated, as was the corresponding mean diversity and heterozygosity (Table 3). There were no apparent differences when the Australian isolates from *A. cerana* were compared to *A. mellifera*, suggesting free transmission of the microsporidian from one host to the other. Accordingly, these two types of isolates were pooled together for further comparisons.

**Fig. 3:**
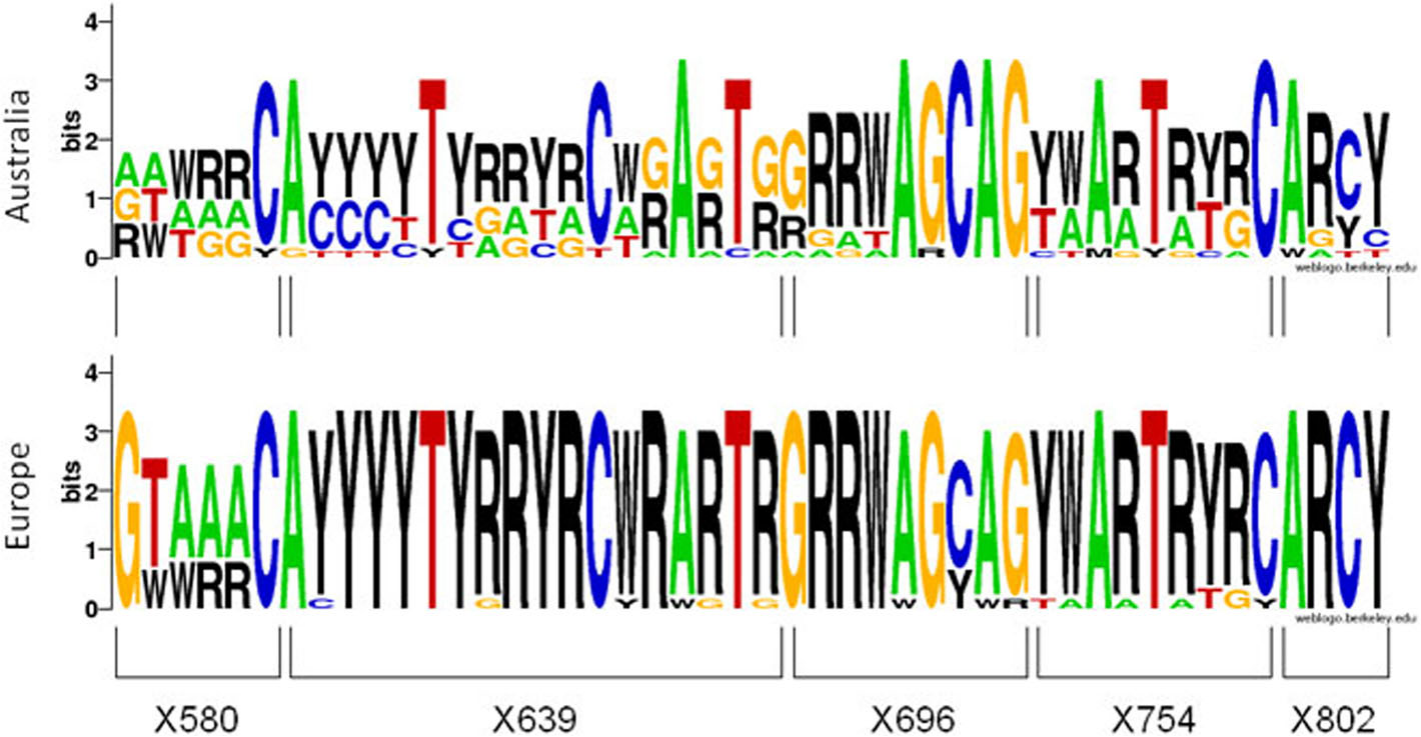
Sequence logo showing the genotype diversity for each polymorphic site. The Australian results are given above and European ones, below

When European isolates were compared to those from Australia, the diversity per site was also similar (Table 3), although more dissimilar genotypes were detected in the latter (the mean numbers of genotypes were 2.8 and 6.6, respectively: t = 3.62, d.f. = 8, *p* < 0.01). Identical genotypes were often found among European samples, not only for a specific marker but also, when considering the five markers. For instance, the genotype 5-11-3-5-3 was obtained from an isolate from Switzerland, another from Slovakia and a third from Spain (Fig. 1), whereas no pair of isolates from Australia shared the same genotype. For most of the marker sites analyzed, the European populations were dominated by heterozygotes while the different genotypes are more balanced in Australia (Fig. 3).

## Discussion

Outbreaks of parasites are typically produced by specific virulent strains. Many parasitic microorganisms reproduce rapidly in an asexual manner and even for those species where a sexual cycle is possible, reproduction is usually clonal [35]. Consequently, it should be possible to identify defined genotypes in a given infection, and assessing their similarity to other genotypes allows epidemiological relationships to be established. In terms of Microsporidia, this is indeed the picture for *Encephalitozoon* species and *Enterocytozoon bieneusi*, with diversity among strains contrasting with the uniform genotypes obtained from specific isolates [36–38]. Yet curiously, these asexual species have been shown to carry out an extant sexual cycle, although it must occur during co-infection of the host by two genetically distinct strains to produce genetic diversity [9]. In these infrequent cases, favorable mutations can arise in separate linages and become combined in the same individual, providing an adaptive advantage to varying environments [35].

In *Nosema* species, genetic evidence clearly supports the existence of cryptic sex in *N. granulosis* [8]. We previously demonstrated that rDNA varies within a given isolate in *N. ceranae* and that recombination may occur, hypothetically between the different repeats of rDNA clusters in sister chromatid exchanges or in a cryptic sexual cycle [18]. This invalidates the use of phylogenetic approaches based on rDNA sequences. In order to eliminate the possibility of exchange between tandem repeat sequences as a source of genetic variability, we selected five putative coding sequences from the genome of *N. ceranae* to study here.

### Diversity and heterozygosis

The results obtained fit into the general picture of strong molecular diversity in *N. ceranae* and a high percentage of shared polymorphisms between different isolates [26]. It is important to emphasize that all five markers analyzed were polymorphic due to single nucleotide changes, ranging from 4 variable sites (in the 875 bp of X802) to as many as 18 (in the 1280 bp of X639). The rates of synonymous versus non-synonymous mutations were fairly close to 1 for X696 and X754, suggesting essentially neutral effects, whereas this rate was clearly biased to synonymous mutations in X639 (Ka/Ks = 0.14), evidence of a purifying selection (that is, most non-synonymous mutations are deleterious and disappear). Similar results were obtained for nine other coding sequences in *N. ceranae* [23]. By contrast, all the polymorphisms detected in the other two markers, X580 and X802, led to changes in the corresponding polypeptide, strongly suggestive of directional selection and a possible adaptive role for these polymorphisms.

Genotyping of the different *N. ceranae* isolates systematically produced ambiguous results as two alternative nucleotides exist at a number of points, which is not a technical artefact. First, indeterminate forms exist for very specific nucleotides. For a given point where a genotype shows a double peak – say, A and G – some other genotypes exhibit a clean A, and other a clean G. Secondly, the cloning and subsequent analysis of different clones demonstrated that the double peaks actually corresponded to alternative DNA templates in a given isolate. Thus, the genotyping of *N. ceranae* has to be directed so as not to obtain a single haplotype corresponding to a specific pure strain but rather, to a whole-isolate genotype that may be of mixed heterozygosity. This heterogeneity can be explained by two different but not exclusive phenomena: (1) a given honey bee is infected by two or more strains of *N. ceranae* and therefore, the isolate includes genetically different clones; and (2) a given strain of the microsporidian has more than one different genome and thus, it might be heterozygous for a number of markers.

### Evidence for recombination

Recombination *per se* is not absolute proof of sex, because clonality does not mean the total absence of it. As recombination is too rare to break the prevalent clonal population pattern [39], a good signal of an asexual reproduction model is a strong linkage disequilibrium (LD) together with a clear phylogenetic signal. By contrast, recombination, high levels of heterozygosis and sustained diversity provide strong evidence of sexual reproduction. Despite the extensive heterozygosity in the genotypes in the isolates, our results provide consistent evidence that different haplotypes can be formed after recombination between two other haplotypes. For instance, clear X580 haplotypes were GTAAA and AATGG, and if both are present in an isolate they will produce the RWWRR genotype, found in a number of isolates. However, AAWRR and GTWRR haplotypes were also found, indicating that AAAAA and GTTGG must also exist (these inferences are detailed in Table 5 and additional examples can be seen in Fig. 1).

**Table 5:**
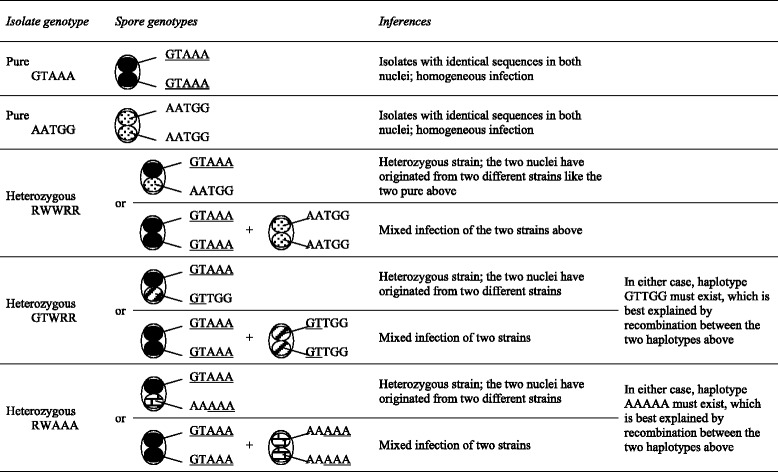
Inferences from the genotypic nature of single spores and isolates

The inferences are based in the most simple model of two haploid nuclei per spore. The example correspond to the results of X580 marker

Moreover, the X639 clones analyzed provide even clearer evidence for recombination. Four clean genotypes were obtained from the Australian samples and genotype 11, the one with most double peaks, was selected for cloning. This genotype could correspond to a mix of genotypes 2, 3 and 4 and the 38 clones sequenced produced 15 different haplotypes: four clones had one of the genotypes 2, 3, or 4; nine were other different recombinants of these and unexpectedly, two clones had a genotype 1. This latter genotype apparently did not exist in the original sample, at least with sufficient prevalence as to produce identifiable peaks in the chromatograms. Thus, direct sequencing from the original isolates highlights the haplotypes existing at considerable frequencies, while minority sequences may not be detected unless many clones are analyzed. Indeed, an earlier analysis of a series of clones indicated that the true allelic richness was most probably underestimated [23].

Strong LD has been found for short reads but those below 200 bp may be too short to identify breaking haplotypes, and LD may decay over larger distances [26]. In fact, our findings support that hypothesis: for example, a pair of nucleotides 27 bp apart is always CC or TT, yet all possible combinations exist with respect to the C or T 291 bp downstream (shadowed in Fig. 2). Thus, to detect recombination longer undivided DNA fragments must be analyzed.

In summary, it appears that *N. ceranae* does not fit into the model of strict asexual reproduction outlined above but rather, the data obtained support the existence of a cryptic sexual cycle.

### Ploidy and diversity in *N. ceranae*

Whatever the mechanisms creating genotype heterogeneity, the fact that all the isolates except one appeared to be mixed or heterozygous for the different markers raises a number of fundamental questions. The *Nosema* genus is binucleate and recent studies even suggest a possible polyploidy for *N. ceranae* [26], with initial discussions focusing on whether nuclear fusion occurs or not, followed by meiotic chromosome reduction [40]. Yet the more important issue here is that the two nuclei must be genetically different in order to drive recombination. The fact is that *N. ceranae* is diverse and recombinants appear systematically. Keeping in mind that millions of spores are produced clonally in a few days, the fact that diversity is maintained throughout the world is baffling. Even a hypothetical existence of two diploid nuclei (2n + 2n spores) as the basis of variability, fusion and meiosis would also be required to sustain the high diversity reported here. However, to explain the variants found at low frequencies, a sexual cycle must also exist independently of the ploidy of each nucleus.

### Absence of geographical barriers

Half of the isolates of *N. ceranae* in this study were obtained from six different European countries, while the other half correspond to Australian isolates from either the European or Eastern honey bee. It is clear that there are no diagnostic alleles specifically related to any of those origins, and except for a few insignificant cases where a variant is found in one single sample, most alleles exist in *N. ceranae* infecting *A. cerana* or *A. mellifera*, from Australia or Europe. The lack of barriers to the transmission of *N. ceranae* between the two honey bees has been reported previously [41] and confirmed recently [23]. Our results indeed point towards free transmission of this microsporidian with no signs of geographical or host segregation. It should be remembered that drones may transmit the parasite to many different honey bee colonies and that *N. ceranae* also infects the *Bombus* species [42, 43], increasing its possibilities of spreading. Additionally, this microsporidian can also be transmitted through pollen [44] and moreover, queens are now freely imported over long-distances to recover honey bee populations in a given region. Thus, a common origin may be associated with different genotypes and indeed, identical genotypes are found in Australia and Europe.

The Eastern origin of *N. ceranae* and the recent invasion into Europe could be associated to founder effects and hence a much lower diversity in Europe than in Australia. Interestingly, the genotype distribution in the samples from the two continents is quite similar - we obtained mean diversities of 0.27 and 0.33, respectively, which is not a significant difference. It is clear that European populations are not of unique origin, as a number of very different genotypes do indeed exist. Heterozygosity and diversity would be related in a population that fits Hardy-Weinberg equilibrium, otherwise the two phenomena may produce different outcomes. Even assuming some degree of sexual reproduction, Hardy-Weinberg expectations would certainly not be expected for an organism like *N. ceranae*. Heterozygosis or mixed genotypes do not parallel its diversity and despite the fewer genotypes in Europe, heterozygosis is not statistically lower (in fact it may be slightly higher). Although further experiments must be performed, this finding again supports an adaptive role for the polymorphic infecting *N. ceranae* populations. The co-existence of different alleles for any genetic marker is maintained worldwide and especially in European isolates. Perhaps, as has also been suggested for *N. bombycis* [45], it is precisely such heterogeneity that makes a population of *N. ceranae* spores so virulent to *Apis mellifera*.

## Conclusions

**Fig. 4:**
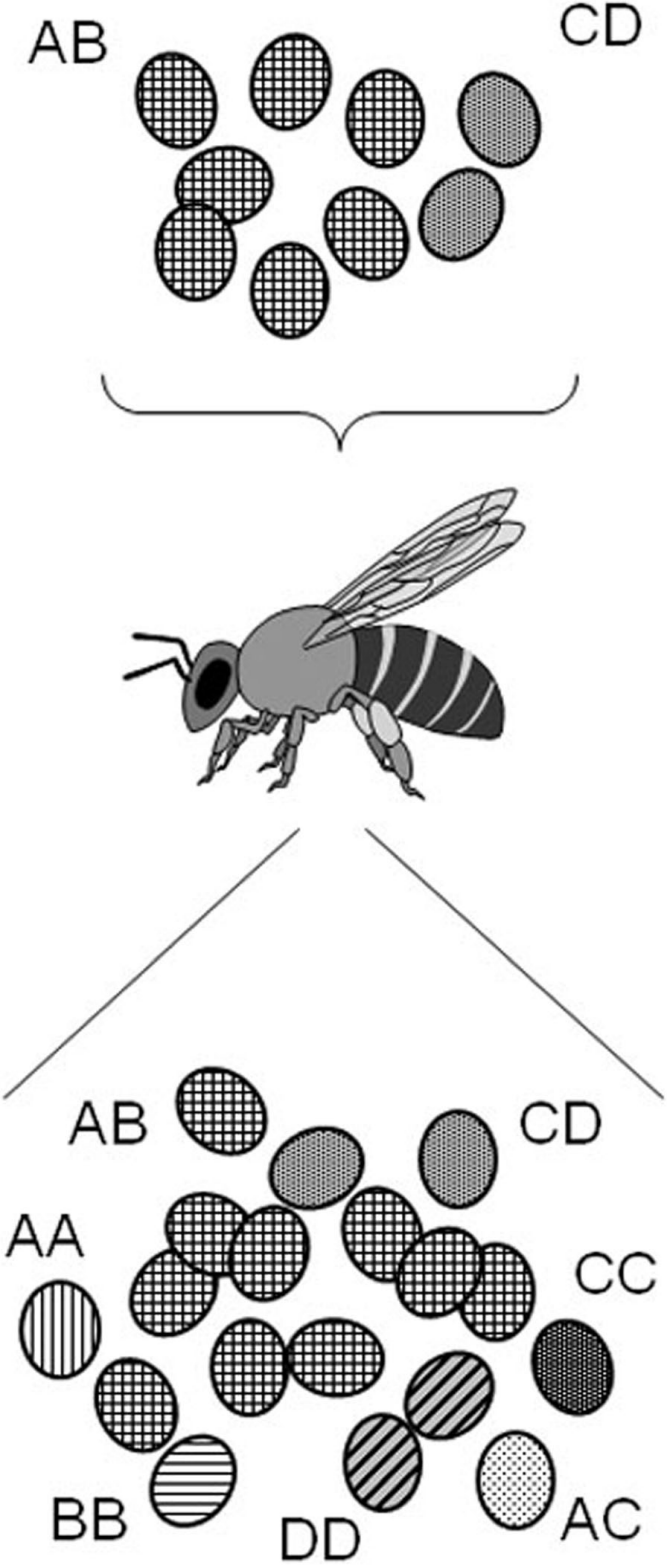
Scheme of genotype diversity generation after multi-strain infection. Up: A honeybee is infected by an heterogeneous collection of *N. ceranae* spores in two possible ways: diploid or pseudodiploid spores may be heterozygous for a given marker (AB), and two or more different strains may be present in the infective group of spores (AB, and CD). Bottom: Most new formed spores may be fully asexual and hence show the same genotypes as above; however, after an hypothetical diploid stage and meiosis, homozygous spores are also formed (AA plus BB in one case, and CC plus DD in the other); additionally if two nuclei of different origin become included in a same spore, new genotypes would also appear (AC and so on). Without sexual reproduction, multi-strain infection could never produce AC, AD, BC or BD haplotypes

The data presented here indicate that honey bees are often infected not by a pure strain of *N. ceranae* but rather, by a heterogeneous group of spores. This may either be because the binucleated spore is heterozygous at a number of loci or because different strains are actually present in the infecting population. As shown in Fig. 4, once this population reproduces in the honey bee gut, meiosis will cause allele segregation and the fusion of nuclei of different origins by recombination also generates new genotypes, maintaining or even augmenting the genetic diversity of *N. ceranae*.

The presence of *N. ceranae* in any region cannot be considered as an outbreak of a specific strain. Wherever sampled, *N. ceranae* populations are diverse, revealing lineages of different origins. Like any organism with an opportunity for sexual reproduction, population approaches must be employed to understand their particular features. The relative ease with which *N. ceranae* has spread worldwide in honey bee colonies reflects the synergistic interaction of mixed infections and the creation of diverse populations of the microsporidium through recombination.
